# Rhythmic categories in horse gait kinematics

**DOI:** 10.1111/joa.14200

**Published:** 2025-01-15

**Authors:** Lia Laffi, Félix Bigand, Christian Peham, Giacomo Novembre, Marco Gamba, Andrea Ravignani

**Affiliations:** ^1^ Department of Human Neurosciences Sapienza University of Rome Rome Italy; ^2^ Department of Life Sciences and Systems Biology University of Torino Turin Italy; ^3^ Neuroscience of Perception and Action Lab Italian Institute of Technology Rome Italy; ^4^ Department of Companion Animals and Horses Movement Science Group, University Clinic for Horses, Vetmeduni Vienna Vienna Austria; ^5^ Center for Music in the Brain, Department of Clinical Medicine Aarhus University and the Royal Academy of Music Aarhus/Aalborg Aarhus Denmark

**Keywords:** bioacoustics, integer ratios, isochrony, locomotion, rhythm

## Abstract

Anecdotally, horses' gaits sound rhythmic. Are they really? In this study, we quantified the motor rhythmicity of horses across three different gaits (walk, trot, and canter). For the first time, we adopted quantitative tools from bioacoustics and music cognition to quantify locomotor rhythmicity. Specifically, we tested whether kinematics data contained rhythmic categories; these occur when adjacent temporal intervals are categorically, rather than randomly, distributed. We extracted the motion cycle duration (t_k_) of two ipsilateral hooves from motion data of 13 ridden horses and calculated the ratios from two successive t_k_ values. We tested whether these ratios significantly fell within rhythmic categories and quantified how close they were to small‐integer ratios, a rhythmic feature also present in animal vocalizations and human music. We found a strong isochronous pattern—a 1:1 rhythmic ratio, corresponding to the ticking of a clock—in the motion of single limbs for all gaits. We also analyzed the interlimb coordination of the two ipsilateral hooves' impacts to identify differences associated with the biomechanical patterns of the three gaits. We found an interlimb 1:1 rhythmic pattern for trot and 1:3 and 3:1 rhythmic categories for walk and canter. Our findings are a first step toward quantifying rhythmicity in horse locomotion and potentially the resulting rhythmic sounds, with possible implications as tools to detect gait irregularities. Overall, we show that rhythmic categories are a valuable tool for gait kinematic analysis and that they can be used to quantify temporal patterns in the motor domain.

## Graphical abstract

Walk, trot, and canter are among the most common gaits in horses. We find that they are all markedly rhythmic, but they differ in more fine‐grained rhythmic and temporal properties.

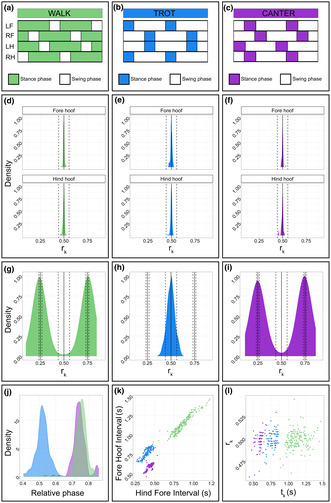

## 1 INTRODUCTION

Rhythms, that is, patterns of events in time, characterize various aspects of an animal's life, from physiology and ecology to behavior (Bass & Baker, 1997; Rusak & Zucker, 1975). Rhythmic movements can act as visual displays, such as the head‐bobbing of green iguanas (Dugan, 1982), the tap‐dancing of cordon bleu birds (Ota, Gahr, & Soma, 2018), and the tail wagging of white wagtails and dogs (Leonetti, Cimarelli, et al., 2024; Randler, 2006). However, we can identify three notable cyclic actions with strong rhythmicity shared by all terrestrial mammals: mastication, breathing, and locomotion (Bramble & Carrier, 1983; Butler et al., 2014; Gerstner et al., 2011; Ghazanfar, 2013; Granatosky et al., 2019). Of these three, locomotion might have a link with other, perhaps cognitive, rhythmic behaviors: the fetus may be acoustically primed by the sound of the mother's gait while in utero (Larsson, 2012, 2014; Larsson et al., 2019).

During mammalian locomotion, the sequence of movements of the limbs is associated with the production of rhythmic sounds: movement and sounds are strictly interconnected (Leonetti et al., 2023; Leonetti, Ravignani, & Pouw, 2024), both theoretically and methodologically. Given the strong association between movement and ensuing sound, the rhythmicity of locomotion can be studied through both kinematic and acoustic approaches. Here, for the first time, we apply methodologies commonly used in animal acoustics — rhythmic categories and small‐integer ratios to kinematics, aiming to understand the physical mechanisms responsible for generating rhythmic sounds. Notably, we will focus on the motor rhythmicity of horses since they exemplify the use of four natural gaits—walk at low speeds, trot at moderate speeds, and canter and gallop at higher speeds.

Locomotion seems intuitively rhythmic; it involves the musculoskeletal system to generate regular and repeated movements over time (Hildebrand, 1965). Animal locomotion features several levels of coordination and rhythmicity. It is characterized by rhythmic coordinated movements of the limbs (Balter & Zehr, 2007; Beer & Chiel, 2002; Ledberg & Robbe, 2011) and other body parts, such as the head (Davies & Green, 1988) or tail (Charrier & Cabelguen, 2013). Moreover, individual limbs must be coordinated in their movement with the other limbs, and this interlimb coordination depends on the specific gait (Danner et al., 2016). In other words, the movement of a limb depends on the coordination among its elements (Ijspeert, 2008). Still, when the animal moves on an even terrain, each limb moves regularly and rhythmically over time, ideally isochronously, that is, characterized by evenly spaced events over time, meaning with a constant duration of successive locomotor cycles (Grillner, 1981). This is especially true for domestic horses that move on racetracks and riding arenas.

The rhythmic quantification of equine gait is essential to describe horse gait kinematics and also to detect horse injuries. Regularity in locomotion is traditionally considered a sign of health (Lewczuk & Maśko, 2021), and rhythmicity of horse gaits is a key element of particular equestrian disciplines, such as dressage (Hobbs et al., 2020). Sometimes, horse gaits may be irregular, and the identification of those irregularities is commonly used to detect lameness. For instance, the stance duration of the lame limb is reduced in the trot (Keegan, 2007; Shrestha et al., 2017; Weishaupt et al., 2001). Moreover, decreased gait symmetry and regularity are symptoms of a horse's state of fatigue, whose recognition is necessary to mitigate injury risk (Lewczuk & Maśko, 2021; Weishaupt et al., 2001). Given the importance of rhythmic evaluation in both kinematics and veterinary fields, quantitative tools to quantify rhythmicity are essential.

Previous studies have quantified interlimb coordination (Drevemo, Fredricson, et al., 1980), stride duration, and other stride characteristics of walk (Matsuura et al., 2003; Nicodemus & Clayton, 2003; Starke et al., 2012), trot (Drevemo, Dalin, et al., 1980; Holmström et al., 1994; Matsuura et al., 2003; Starke et al., 2012), and canter (Back et al., 1997; Ratzlaff et al., 1995; Splan & Hunter, 2004). These studies analyze the regularity of different gaits by measuring temporal kinematic parameters (e.g., stance, swing, stride durations) and their variation; they measure and report, for example, coefficients of variation (e.g., Drevemo, Dalin, et al., 1980) and standard deviation (e.g. Drevemo, Dalin, et al., 1980; Ratzlaff et al., 1995). Such metrics quantify some regularities in the sequence of movements that characterize the motor temporal pattern. These are distributional metrics, which cannot capture the structure among intervals. Recently, rhythmic quantification has increasingly focused on analyzing relationships between two or more adjacent intervals in a temporal sequence (Ravignani & Madison, 2017; Roeske et al., 2020): here, for the first time, this approach is applied to quantify locomotor rhythmicity. To better understand this approach to rhythmic quantification, a clock exemplifies a rhythm with intervals demonstrating a 1:1 relationship, where each tick occurs at a regular and equal temporal distance from the previous one. Anomalies in a hypothetical clock would be only partially detected in temporal analyses using coefficients of variation and standard deviation: If the clock skips a tick every three ticks, these analyses will show a generic higher variation. However, rhythmic analyses that consider relationships between adjacent intervals would be able to clearly detect those anomalies through the emergence of ratios other than 1:1. Along these lines, to have a rhythmic pattern, successive intervals do not need to have the same duration; it is sufficient that the relationship between them is repeated in the time sequence. In brief, rhythmic analyses are meant to complement and enhance, rather than replace, classic kinematic analyses.

**FIGURE 1:**
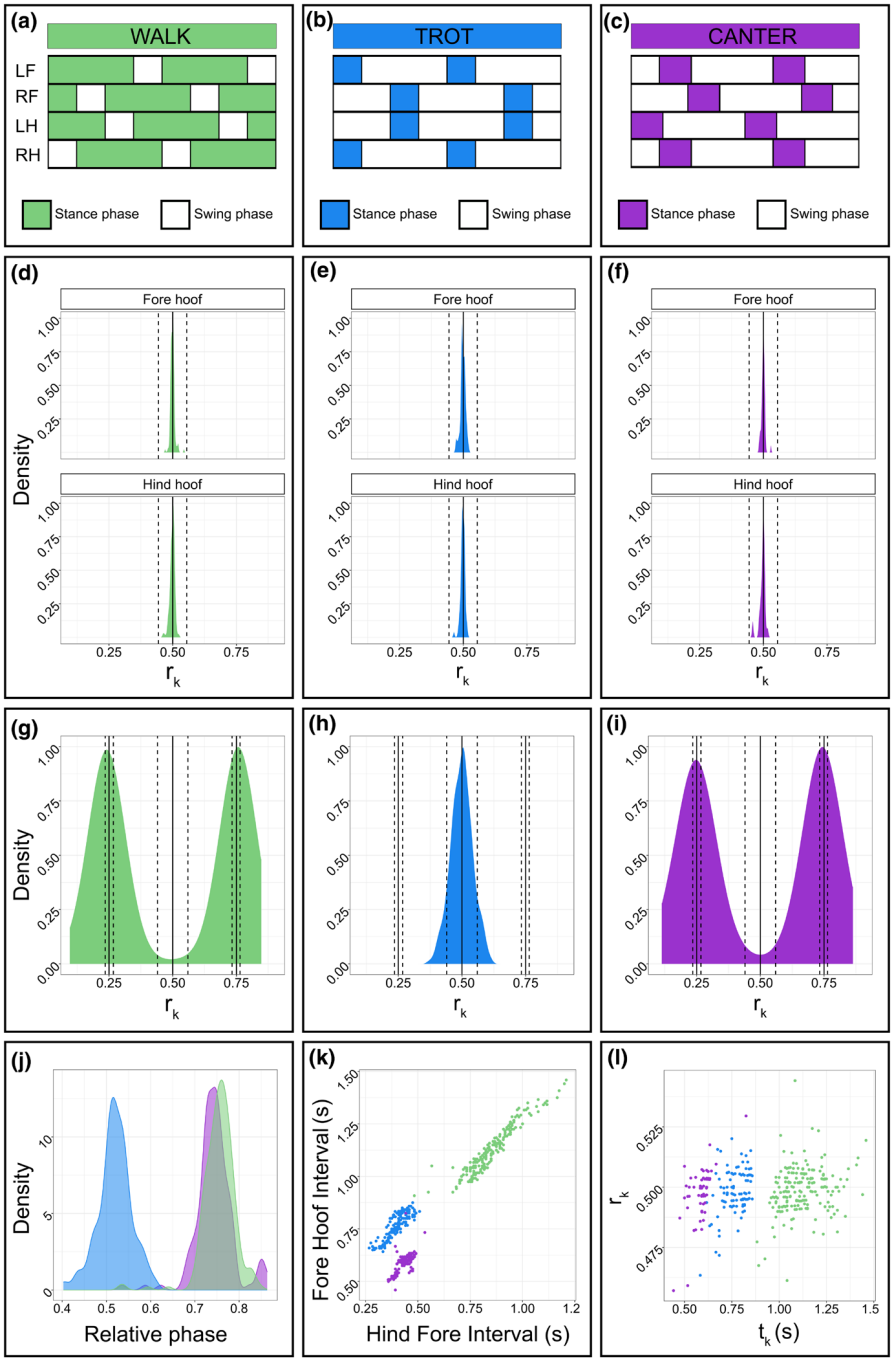
Rhythmic structure of horse gaits: Walk in green, trot in blue, and canter in purple. (a–c) Gait diagrams. The hoof impact defines the beginning of the motion cycle, which consists of a stance phase, where the hoof is in contact with the ground (colored sections), and a swing phase, where the hoof is off the ground (white sections). For this study, we considered the right fore hoof and the right hind hoof. (d–f) Probability density functions representing the distribution of integer ratios (*r*_*k*_) calculated from Fore Hoof Intervals (FHI) and Hind Hoof Intervals (HHI). The black lines correspond to perfect isochrony, and the dotted lines delimit the corresponding on‐integer ratio ranges. (g–i) Probability density functions representing the distributions of integer ratios (*r*_*k*_) calculated from Inter Hoof Intervals. The black lines correspond to perfect small‐integer ratios (1:3, 1:1, and 3:1), and dotted lines delimit the corresponding on‐integer ratio ranges. (j) The probability density function of the relative phase is the ratio between Inter Hoof Intervals and Fore Hoof Intervals. (k) Scatterplot of Fore Hoof Intervals against Hind Fore Intervals, or the interval between a hind hoof impact and the successive fore hoof impact. (l) Scatterplot of t_k_ values for both Fore Hoof Intervals and Hind Hoof Intervals, against their corresponding *r*_*k*_ values.

The concept of rhythmic categories is derived from human music, where the durations of adjacent intervals are not only categorically distributed but are also (sub)multiples of each other (Van den Bosch Der Nederlanden et al., 2023). Specifically, adjacent intervals tend to show relationships corresponding to small integer ratios (e.g., 1:1, 1:2, 1:3). Rhythmic categories emerge when a ratio based on small integers is repeated in a sequence. For instance, if consecutive intervals in a musical sequence consistently display a 1:2 ratio, a rhythmic category of 1:2 emerges. Although originally developed to describe human music, the concept of rhythmic categories can be applied to any temporal sequence. These ratios have recently been found in the vocalizations of some mammals and birds (e.g., De Gregorio et al., 2021, 2024; Lameira et al., 2023; Raimondi et al., 2023; Roeske et al., 2020). In this study, we apply rhythmic categories to the field of kinematics to test if horses' locomotor movement is “rhythmically quantized” in time according to small‐integer ratios. We analyzed the movement of single hooves in three natural horse gaits—walk, trot, and canter—and the interlimb coordination patterns of two ipsilateral limbs, aiming at demonstrating rhythmicity in the movement of single limbs and probing differences in interlimb coordination patterns across gaits. We hypothesize a strong rhythmicity in the movement of single limbs. Specifically, we expect the emergence of an isochronous pattern (1:1 rhythmic category) in the movement of a single limb, that is, we expect motion cycles to have the same duration, where each cycle corresponds to the time between consecutive hoof impact events and is defined by the sum of stance and swing phase of the limb (Figure 1a–c). Regarding the interlimb coordination patterns, we expect a strong rhythmicity of interlimb patterns with some differences among gaits. The trot is a two‐beat gait with a simultaneous movement of diagonal pairs of limbs, the walk is a four‐beat gait, and the canter is a three‐beat gait with a suspension phase. We then expect an isochronous interlimb rhythmic pattern for the trot (1:1), meaning that the impacts of two ipsilateral hooves will generate intervals of equal duration. We expect the presence of rhythmic categories other than isochrony in walk and canter; in particular, we expect to find the 1:3 and 3:1 rhythmic categories because the ipsilateral limbs move in successive phases of the motion cycle in both walk, characterized by four‐foot impacts per cycle, and canter, characterized by three impacts followed by suspension phase.

## 2 MATERIALS

Our analyses focus on the rhythmicity of walk, trot, and canter. The *walk* (Figure 1a) is a four‐beat gait in which all the limbs impact the ground independently and with no suspension phase. The walk is a diagonal gait, where the right foreleg is followed by the left hind and left fore, followed by the right hindlimb. The *trot* (Figure 1b) is a two‐beat gait in which diagonal pairs of limbs move simultaneously. The trot features two moments of suspension between the impact of each diagonal pair of limbs. The *canter* (Figure 1c) is a three‐beat gait characterized by four phases. The trailing hindlimb makes the first ground contact. It is followed by the landing of the leading hindlimb and trailing forelimb, almost simultaneous but with possible slight dissociations (Clayton, 2016). The leading forelimb then strikes the ground, and a suspension period follows (Hildebrand, 1965; Ross, 2011; Starke et al., 2009).

We used data from 13 horses of various breeds, sex, and age (Data S1) recorded by Witte and colleagues (Witte, Schobesberger, & Peham, 2009). Riders were asked to allow horses to travel at their preferred speed for each gait. The recordings were collected along a straight section of a path, specifically on a 12‐m‐long pressed sand track. All horses were ridden with English Saddle and Side Saddle in walk, trot, and canter. At canter, the right limbs were always the leading limbs. This generates a 2 × 3 (saddle × gait) design (Witte, Schobesberger, & Peham, 2009).

## 3 METHODS

### 3.1 Motion capture recordings

**FIGURE 2:**
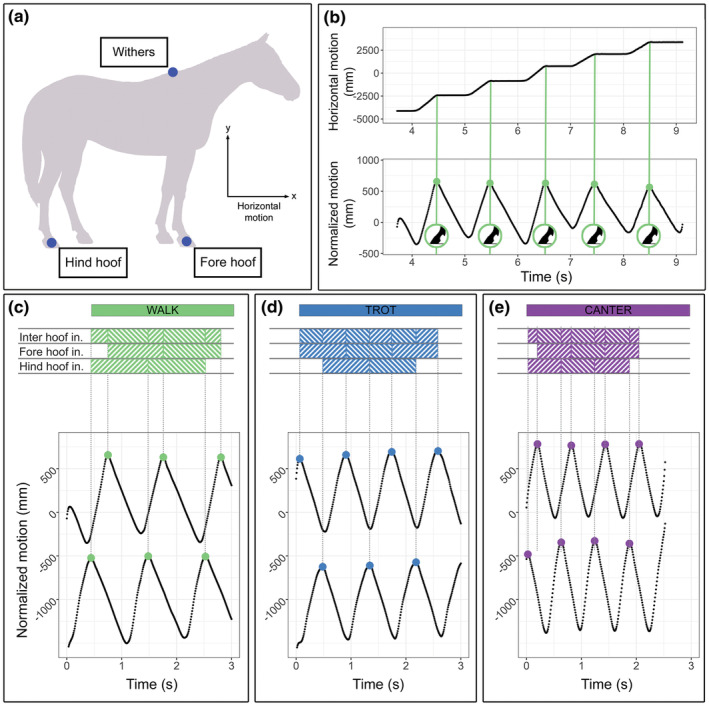
Extraction of time sequences from hooves' sagittal displacements. (a) Marker position: fore hoof, hind hoof and withers. (b) Hoof's sagittal displacement (top) shows the displacement of the hoof as a function of time. The normalized hoof's sagittal displacement (bottom) represents hoof displacement adjusted to the withers position since the withers is the origin of the horse‐based coordinate system. Colored points indicate the hoof impact, marking the beginning of a motion cycle. (c–e) Fore hoof and hind hoof normalized displacement through time. The time sequence of fore hoof and hind hoof impacts defines the Fore Hoof Intervals (FHI), Hind Hoof Intervals (HHI), and Inter Hoof Intervals (IHI).

Three‐dimensional motion capture data were recorded by Witte and colleagues (Witte et al., 2009). Six cameras were placed along the right side of the measurement track, and the marker positions were recorded with a sampling rate of 120 Hz using the Expert Vision System of the Motion Analysis Corporation (Santa Rosa, California). The tracking of the reflective passive markers was semi‐automatic. Our analysis only included the withers, fore hoof, and hind hoof markers on the right side of the horse, for a total of three markers (Figure 2a). In the canter, the limbs considered were the leading limbs. For this study, we only considered displacement in the sagittal plane, that is, the direction of movement of the horse. Additional methodological details of data acquisition are reported by Witte and colleagues (Witte et al., 2009).

### 3.2 Fore and hind hoof interval extraction

Using the software RStudio (RStudio Team, 2020), the time series of the markers were smoothed using a lowpass Butterworth filter (package “seewave,” Sueur et al., 2008) with a cutoff frequency of 15 Hz for hoof markers and 5 Hz for the withers (Witte, Schobesberger, & Peham, 2009). Such coordinates vary relative to the origin of the global coordinate system. To quantify motion rhythmicity irrespective of where the horse was located in the room, we normalized hind and fore hoof positions to the withers, by subtracting the withers' coordinates from the hoofs' coordinates, at each time frame. To calculate the duration of motion cycles, we focused on the hind and fore hoof horizontal displacements on the *x*‐axis, coinciding with the direction of the horse movement (Figure 2a). We extracted the positive peak values of the normalized hoof coordinates of the *x*‐axis (Figure 2b) with R's peakwindow function (package “cardidates,” Petzoldt, Sachse, & Rolinski, 2007). The peak values we calculated correspond to the hoof impact, as they mark the onset of the stance phase of the motion cycle (Figure 2b). The total duration of a motion cycle (t_k_), corresponding to the sum of the stance and the swing phase of the limb (Figure 1a–c), was calculated as the interval between a cycle's maximum peak and the following one's maximum peak. From now on, we will refer to the forelimb cycles' duration as Fore Hoof Interval (FHI) and the hindlimb cycles' duration as Hind Hoof Interval (HHI) (Figure 2c–e).

### 3.3 Testing isochronous patterns: FHI and HHI

We adopted a method used in acoustics to evaluate the occurrence of small‐integer ratios in the FHI and HHI (Roeske et al., 2020). For both FHI and HHI temporal sequences, ratios (*r*_*k*_) were calculated by dividing the duration of each motion cycle (t_k_) by its duration plus the duration of the following one (Roeske et al., 2020): *r*_*k*_ = t_k_/(t_k_ + t_k+1_). We divided the *r*_*k*_ distribution into on‐integer and off‐integer ratio ranges, where the on‐integer ratio ranges are closer to the value of the small‐integer ratio. A small‐integer ratio refers to a ratio between two numbers where both numbers are small integers, for example, 1:1. A ratio (*r*_*k*_) value of 0.500 corresponds to the 1:1 small‐integer ratio, isochrony, which is obtained when consecutive intervals have equal duration. We centered the on‐integer ratio range around 1:1 (*r*_*k*_ = 0.500) and set the boundaries of the on‐integer ratio range between 0.444 and 0.556. We defined the noninteger ratio range between 0.400 and 0.444 and 0.556 and 0.600. These cutoff values coincide with those previously used in the literature (Roeske et al., 2020). For each horse, gait, and saddle combination, we counted the on‐integer and off‐integer ratios calculated from the FHI and the HHI. We compared the count of data points falling in the on‐ versus off‐integer ratio ranges using paired (two‐sided) Wilcoxon signed‐rank tests, one for each combination of hooves (fore vs. hind), saddle type (English vs. side saddle), and gait type (walk, trot, and canter).

### 3.4 Testing accuracy and precision

To test for differences between the isochronous pattern of various gaits and the two types of saddles, we computed two variables capturing different nuances of rhythmicity: deviance and spread of *r*_*k*_ values. For each animal, we calculated the maximum value in the deviance density function—its peak value—for every gait and saddle combination (bandwidth: *bw* = 0.00194663). Deviance measures accuracy, quantifying the degree of proximity of *r*_*k*_ values to ideal isochrony. Deviance was calculated as the absolute value of the distance between *r*_*k*_ and the center of the small integer ratio, that is, |*r*_*k*_ − 0.500|. S*pread* measures precision, quantifying how close the *r*_*k*_ values are to each other. Spread was calculated as the difference between the third and the first quartile for each gait and saddle type, that is, the interquartile range. We created two GLMMs (package “glmmTMB,” Magnusson et al., 2019), one for deviance and one for spread as dependent variables. Beta was chosen via the package *fitdistrplus* as a suitable theoretical distribution (Delignette‐Muller et al., 2023). We entered deviance or spread as the response variables, hoof (FH vs. HH), gait, and their interaction as fixed factors, and the horse identity as a random factor.

### 3.5 Visualizing interlimb rhythmic patterns

To visualize the rhythmic pattern of each gait, we calculated integer ratios based on the impact sequence of the two ipsilateral hooves, the Inter Hoof Intervals (IHI) (Figure 2c–e). These ratios were then plotted in a density graph.

### 3.6 Testing relative phase

We calculated the relative phase representing the shift between fore and hind hoof impacts. This was the ratio between the Hind Fore Interval (HFI), defined as the time lag between the hind hoof impact and the following fore hoof impact (*HFI* = *HHI – FHI*), and the FHI: *Relative phase = HFI*/*FHI*. We used a GLMM with beta distribution to test for relative phase differences between different gait and saddle types. Beta was chosen via the package fitdistrplus. We entered relative phase values as the response variable, gait as a fixed factor, and horse identity as a random factor.

### 3.7 Testing the relationship between FHI and HFI

FHI and Hind Fore Intervals are influenced by different parameters: FHI by instantaneous gait tempo, hind fore intervals by both tempo and interlimb coordination pattern of the ipsilateral hooves. For this reason, studying their relationship may help identify differences among gait rhythmic patterns. We also created a GLMM to test the relationship between the FHI and Hind Fore Intervals. We considered the FHI as the response variable and the Hind Fore Intervals, gait, saddle, and their interactions as fixed effects. We used the horse identity as a random effect. The GLMM fitted a beta distribution chosen via the package *fitdistrplus*.

### 3.8 GLMM models: Additional information

We chose GLMM models through model selection. We compared models' Akaike Information Criterion (AIC) values with different fixed factor combinations and chose the lowest value (Sakamoto et al., 1986). To test the significance of each model, we compared it with a null model that included only the random factor and offset, using a likelihood ratio test (Dobson, 2002). Using the R summary function, we obtained *p*‐values for each predictor. With the emmeans package, we obtained pairwise comparisons (*p*‐values adjusted for multiple comparisons with the Tukey method) (Lenth et al., 2022). We verified the normality and homogeneity of residuals by inspecting the qqplot and the residuals' distribution for each model. All statistical analyses were performed with RStudio.

## 4 RESULTS

### 4.1 Isochronous patterns: FHI and HHI

For both the fore and the hind hooves, and any type of saddle and gait, from Wilcoxon signed‐rank tests, we found that significantly more integer ratios were categorized into the isochronous on‐integer range compared to those categorized as isochronous off‐integer (all W ≤ 1.5, all *p* < 0.001). Gait isochrony means that consecutive motion cycles have the same duration, both for the sequence of FHI and HHI. Specifically, all the *r*_*k*_ values calculated from the FHI and the HHI fall within the on‐integer ratio range. These results suggest an extreme rhythmicity of the limbs in all the different gaits we analyzed (Figure 1d–f). Additionally, Figure 1l highlights that, regardless of the different interval values produced by each gait, both FHI and HHI are associated with very similar *r*_*k*_ values.

### 4.2 Accuracy and precision

GLMMs on deviance and accuracy revealed differences across gaits in the accuracy (deviance) of motor rhythmicity of the fore hoof, namely how close the *r*_*k*_ values are to perfect isochrony, but no differences in precision (spread) (Laffi et al., 2024). Deviance (Data S1) is influenced by gait, hoof, and their interaction (Full vs. Null: *χ* ^2^ = 36.868, df = 11, *p*‐value <0.001). In the trot, the fore hoof exhibits greater deviance than the canter (*z* = −3.662, *p* = 0.003) and walk (*z* = 4.489, *p* < 0.001). Furthermore, the deviance of the trot of the fore hoof is higher than that of the hind hoof (*z* = 3.483, *p* = 0.006). Spread measures precision, quantifying how close *r*_*k*_ values are to each other. The spread (Full vs. Null: *χ* ^2^ = 23.53038, df = 11, *p*‐value = 0.015) is influenced by gait and hoof, but no differences are detected in pairwise comparisons (Data S1).

### 4.3 Interlimb rhythmic patterns

To visualize the rhythmic pattern of each gait, we plotted integer ratios based on Inter Hoof Intervals (IHI) (Figure 2c–e). In the density plot, we can see a clear single peak for the trot at around 0.500, corresponding to isochrony, and two peaks for the other walk and canter, corresponding to the 1:3 (*r* = 0.25) and 3:1 (*r* = 0.75) rhythmic categories (Figure 1g–i). Considering the hooves' impact sequence, we can explain the isochronous peak of the trot with intervals between hooves' impacts having equal durations. To illustrate the two peaks of the walk and canter, if the total duration of the movement cycle is 4 time units, then 1 time unit will separate the impact of the hind hoof and the fore hoof, and 3 units will occur between the fore and hind impacts.

### 4.4 Relative phase

In the quantitative analysis of the relative phase, the ratio between the Hind Fore Interval and the FHI (Full vs. Null: *χ* ^2^ = 1258.103, df = 1, *p*‐value <0.001) shows differences across gaits (Data S1); the highest values occur at walk (walk vs. canter, *z* = −2.860, *p* = 0.002; walk vs. trot, *z* = −69.084, *p* < 0.001) and lowest ones at trot (walk vs. canter, *z* = 58.934, *p* = 0.002). Figure 1j visualizes the differences found in the model via a density plot containing three distinct peaks, one for each gait. The trot has a maximum peak value of 0.515, the walk at 0.760, and the canter at 0.744. This result and visualization suggest that walk and canter can be differentiated from the rhythmic movement pattern in ipsilateral limbs.

### 4.5 Relationship between FHI and HFI

The GLMM model (Full vs. Null: *χ* ^2^ = 1036.630, df = 10, *p*‐value <0.001) to test the relationship between FHI and Hind Fore Interval shows a higher slope for trot than for canter or walk (all *p* < 0.001) (Data S1). In other words, no matter the saddle, similar values of Hind Fore Intervals are associated with higher Fore Hoof Intervals in the trot than in the canter and walk. Plotting FHI versus Hind Fore Interval delivers three distinct clusters of points, one for each gait (Figure 1k). The FHI interval equals the instantaneous gait tempo, representing the time between successive hoof impacts of a single limb. In contrast, Hind Fore Intervals are influenced by the interlimb rhythmic pattern and the overall gait tempo. The plot shows that walk has the highest Fore Hoof and Hind Fore Intervals values. Canter and trot exhibit similar Hind Fore Intervals, but the Fore Hoof Intervals for trot are higher. It is evident that the walking pace is slower than the trot, and the trot is slower than the canter.

## 5 DISCUSSION

This study aimed to examine the rhythmic regularity of quadrupedal locomotion, specifically by identifying rhythmic categories within the kinematic data of three different horse gaits. We considered the movement of single limbs and the pattern of interlimb coordination of two ipsilateral limbs to dissect the building blocks of gait rhythms. We found that the movement of both fore‐ and hindlimb is rhythmic, and specifically isochronous—like a ticking clock—with similar precision values but different accuracy values across gaits. Furthermore, despite the different load distribution these two saddles impose on the equine back, the rhythmic pattern did not differ significantly when measured in horses ridden in a side‐saddle or general (astride) English saddle (Winkelmayr et al., 2006). These results fully confirm our first hypothesis, complementing and extending both layperson knowledge of horse gait and previous studies that found a strong regularity in the motion of horses' limbs (e.g., Drevemo, Dalin, et al., 1980; Hodson et al., 1999), with no differences between saddle types (Ramseier et al., 2013).

The isochronous pattern we find may have both functional and evolutionary explanations. Physiologically, locomotion is a complex behavior that depends on specific neural circuits, namely central pattern generators responsible for producing rhythmic and coordinated motor patterns. Additionally, sensory feedback mechanisms allow the adjustment and optimization of the movement (Grillner & El Manira, 2020). From an evolutionary perspective, two main pressures may lead to strong rhythmicity in gait. First, rhythmicity is related to reducing the amount of energy used during locomotion (Ross, 2011) since greater interstride variability is associated with increased energy consumption (Granatosky et al., 2018; O'Connor et al., 2012). Second, the isochronous movement of the limbs we found is also justified by the possibility of anticipating specific movements during locomotion, as suggested by Faltings, Young, Ross, and Granatosky (2022). Motion predictability is essential to coordinate oscillations in musculoskeletal and nervous systems, reducing the probability of interlimb interference and falls that are usually linked to interstride variability (Larsson, 2014; O'Connor et al., 2012; Ross et al., 2013). In brief, the isochronous pattern observed in the three gaits provides an energetic advantage and improves limb coordination, supporting highly efficient equine locomotion.

Each gait is generally used within a limited speed range: it has been shown that, at a nonpreferred speed of a given gait, interstride variability tends to increase, driving animals into more variable and unstable locomotor conditions that can lead to interlimb interference or falling (Granatosky et al., 2018; Jordan et al., 2007). For this reason, the reduction of interstride variability has also been proposed as one of the possible triggers of gait transition (Granatosky et al., 2018). Our results show that the isochronous rhythmic pattern of trot has lower accuracy than those of canter and walk. The lower regularity we found could be an effect of a higher interstride variability and may be directly linked to greater variability in the animals' speeds while performing the trot. In other words, it is possible that all the horses maintained a speed very close to their preferred speed for walking and cantering. In contrast, when trotting, there were more variations with a consequent increase of the interstride variability. Further research would be needed to verify the relationship between isochronous pattern regularity and speed.

Our results demonstrate that Hind Fore intervals and FHI show a similar relationship in walk and canter, suggesting a similar interlimb coordination pattern. However, Figure 1k shows evidence that both FHI and Hind Fore Intervals are longer in the walk than in other gaits. These results are aligned with previous work, which found that walks have longer motion cycles and stance durations than the other gaits (e.g., Robilliard, Pfau, & Wilson, 2007). Moreover, the walk shows longer intervals between the hind and the fore hoof impacts, which we call the Hind Fore interval, than the trot and the canter. Different speeds partially explain these results: canter and trot are usually faster than walk (Grillner, 1981; Hildebrand, 1989). Conversely, speed does not alone account for rhythm: similar Hind Fore Interval values between trot and canter derive from a combination of different speeds and different rhythmic patterns (Figure 1g–i). The consistency of our results with previous kinematic studies suggests that the methodology adopted here might be a valuable and complementary tool for equine gait analysis.

In agreement with our second prediction, considering the movement of two ipsilateral hooves and their temporal relationships, we have quantified a significantly different rhythmic pattern among walking, trotting, and cantering. We found a relative phase of 0.515 for the trot, and from the *r*_*k*_ sequence of the two ipsilateral hooves' impact, we can see a single peak corresponding to the 1:1 rhythmic category (Figure 1g–i). The relative phase value we quantified is slightly different from the isochrony ideal value, meaning that the interval between the fore and hind hoof impacts is slightly shorter than the interval between the hind and fore hoof impacts. As expected, these results show that ipsilateral hooves, with an almost perfectly isochronous rhythmic pattern, impact the ground at regular intervals (Hildebrand, 1965). In both walk and canter, from the sequence of fore and hind hooves impact, we can see two peaks corresponding to the 1:3 (0.25) and 3:1 (0.75) rhythmic categories (Figure 1g–i). Our results align with previous literature, as the walk is a four‐beat gait in which, ideally, the beats of the four limbs are equally spaced over time (Hildebrand, 1965). In a motion cycle divided into four equal intervals, two ipsilateral hooves strike the ground sequentially, creating two intervals, one lasting three times the duration of the other. The canter, a three‐beat gait, is characterized by three beats followed by a long suspension phase (Hildebrand, 1989). Similar to the walk, the ratios we observed (1:3 and 3:1) suggest that, given a cycle with a total duration of four units, the interval between the impacts of the leading forelimb and hindlimb is three times longer than the interval between the impacts of the leading forelimb and the following forelimb. In agreement with recent research on locomotion‐induced sounds in the canter, our findings suggest that the suspension phase may last twice as long as the other intervals within the motion cycle (Laffi et al., 2024). Even if the locomotor rhythmic patterns appear very similar between the two gaits, just considering rhythmic categories, we found that the phase shift of the ipsilateral limbs of the walk is different from the phase shift of the canter.

Our results confirm that horses exhibit a regular pattern of movement over time, providing for the first time a rhythmic categories‐based quantification of this kinematic phenomenon (Drevemo, Dalin, et al., 1980; Matsuura et al., 2003; Splan & Hunter, 2004; Starke et al., 2012). We have demonstrated the presence of a similar isochronous movement for each limb but a different pattern of interlimb coordination in three horse gaits. As movements and sounds are strictly related, the strong rhythmic pattern we found in horse motion, coupled with differences among various gaits, provides a mechanistic basis to a common human intuition: one can qualitatively distinguish among horse gaits by the sound of hooves (Laffi et al., 2024). Therefore, since the sound is a direct consequence of the movement (Leonetti, Ravignani, & Pouw, 2024), it would be interesting to test the complex mapping between locomotor rhythms and differences between gaits, on the one hand, and the sound of hooves, on the other hand.

We found strong rhythmicity in horse locomotion. However, our study has two major limitations. First, our results are based on the horse's ipsilateral limbs; thus, our results cannot wholly describe the rhythmic pattern of horse gaits. Second, horse locomotion was recorded in a nonnatural context, specifically on even terrain, at constant speeds, with the potential influence of the rider. Nonetheless, this study shows, for the first time, that rhythmic categories and small‐integer ratios can be used to measure locomotor rhythmicity. Future research might employ our methodologies to explore complete rhythmic patterns, considering the movements and symmetry of all four limbs and the impact of particular conditions, such as Diagonal Advanced Placement (Clayton, 1997). Future studies could also investigate how the rhythm of walking, trot, and canter varies under conditions different from those tested here. For instance, one could study rhythm in uneven terrain and at varying speeds or compare gait rhythms with and without the rider. Furthermore, since animals with lameness exhibit reduced motor regularities, rhythmic categories could show potential as a complementary tool for quantifying gait irregularities in a clinical setting; for this, further research is required. In conclusion, considering that most animals share cyclic movements, we hope that the metrics used in this study may be helpful in future kinematic studies, within and across species.

## AUTHOR CONTRIBUTIONS

C.P. designed the original study; C.P. collected the data; L.L. and A.R. designed the study; L.L. performed the analyses; L.L. wrote the original draft; L.L. and M.G. made figures; F.B, G.N., M.G., and A.R. supervised the research and provided input on analyses, writing and figures; all authors reviewed and edited the manuscript.

## CONFLICT OF INTEREST STATEMENT

The authors declare no competing interest.

## Supporting information


Data S1.

## Data Availability

The data used in this study are sourced from Witte et al.'s (2009) paper. Please refer to their publication for information on data accessibility and availability. Witte et al. (2009). Motion pattern analysis of gait in horseback riding by means of Principal Component Analysis. Human Movement Science, 28, 394–405. Available at: 10.1016/j.humov.2009.04.002.
