# Supplementary material for: Rhythmic categories in horse gait kinematics
Source: J Anat. 2025 Jan 15;246(3):456–65. doi: 10.1111/joa.14200 (PMC11828748; doi:10.1111/joa.14200)
Supplement: Supplementary file 1 — Data S1. [file JOA-246-456-s001.pdf]

Rhythmic categories in horse gait kinematics

Supplementary information

Lia Laffi[1,2]*, Félix Bigand[3], Christian Peham[4]*, Giacomo Novembre[3], Marco Gamba[4§], Andrea Ravignani[1,5 §*]

[1] Department of Human Neurosciences, Sapienza University of Rome, Rome, Italy
[2] Department of Life Sciences and Systems Biology, University of Torino, Turin, Italy
[3] Neuroscience of Perception & Action Lab, Italian Institute of Technology, Rome, Italy
[4] Department of Companion Animals and Horses, Movement Science Group, University Clinic for Horses, Vetmeduni Vienna, Vienna, Austria
[5] Center for Music in the Brain, Department of Clinical Medicine, Aarhus University & The Royal Academy of Music Aarhus/Aalborg, Denmark

§Co-senior authors
*Corresponding authors
lia.laffi@unito.it
christian.peham@vetmeduni.at
andrea.ravignani@uniroma1.it

S1 - General information of recorded horses

Horse ID	Gender	Breed	Age	Withers height (m)
**Ali**	Mare	Austrian Warmblood	7	1.80
**Bla**	Mare	Slovakian Warmblood	9	1.60
**Faw**	Mare	Mix (Kladruber and a Welsh)	9	1.58
**Fleur**	Mare	Austrian Warmblood	13	1.63
**Nom**	Mare	Gidran	16	1.63
**Ove**	Gelding	Criollo	8	1.50
**Roc**	Gelding	Austrian Warmblood	16	1.65
**Sav**	Stallion	Lippizaner horse	19	1.64
**Sca**	Gelding	Mix (Arabian and Islandic)	13	1.40
**Sch**	Gelding	Czech Warmblood	8	1.67
**Stu**	Gelding	Haflinger	7	1.42
**Tri**	Mare	Austrian Warmblood	14	1.50
**Xen**	Gelding	Czech Warmblood	25	1.70

S2 - Summary and details of the GLMM and pairwise post-hoc tests, modeling how gait and limb type may affect (rhythmic) deviance.

[a] Summary of the full model - Influence of the fixed factors (Gait: Canter - Trot - Walk; Hoof: Fore - Hind) on the deviance. The horse is the random factor (horse ID).

[b] Results for the pairwise post-hoc test on the interaction gait * hoof

a

Generalized Linear Model

Family: beta

Deviance ~ gait * hoof + (horse ID)

Full vs Null (Chisq = 28.849; df = 5; p < 0.001)

Variable	Estimate	SE	z value	p-value (z)
(Intercept) [b,c]	-5.418	0.195	[a]	[a]
Gait (t) [b,c]	0.819	0.224	3.662	<.001
Gait (w) [b,c]	-0.082	0.242	-0.341	0.733
Hoof (h) [b,c]	0.256	0.241	1.064	0.287
Gait (t): hoof (h) [b,c]	-0.937	0.311	-3.009	0.003
Gait (w): hoof (h) [b,c]	-0.442	0.331	-1.334	0.182

[a] Not shown as not having a meaningful interpretation

[b] Estimate ± SE refer to the difference in the response between the reported level of this categorical predictor and the reference category of the same predictor

[c] Reference categories: “gait (c)”, “hoof (f)”

b

***Post-hoc* comparisons**

Emmeans

Gait	Hoof	emmean	SE	lower.CL	upper.CL
canter	fore	-5.42	0.195	-5.80	-5.04
trot	fore	-4.60	0.132	-4.86	-4.34
walk	fore	-5.50	0.170	-5.83	-5.17
canter	hind	-5.16	0.165	-5.49	-4.84
trot	hind	-5.28	0.162	-5.60	-4.96
walk	hind	-5.69	0.180	-6.04	-5.33

Contrasts

Contrast	Estimate	SE	z ratio	p-value
c f - t f	-0.819	0.224	-3.662	0.003
c f - w f	0.082	0.242	0.341	0.999
c f - c h	-0.256	0.241	-1.064	0.896
c f - t h	-0.138	0.239	-0.578	0.993
c f - w h	0.268	0.248	1.080	0.890
t f - w f	0.901	0.201	4.489	<.001
t f - c h	0.563	0.198	2.841	0.051
t f - t h	0.680	0.195	3.483	0.007
t f - w h	1.087	0.207	5.250	<.0001
w f - c h	-0.339	0.220	-1.538	0.640
w f - t h	-0.221	0.218	-1.011	0.914
w f - w h	0.186	0.227	0.817	0.965
c h - t h	0.118	0.217	0.543	0.994
c h - w h	0.524	0.227	2.307	0.191
t h - w h	0.407	0.223	1.824	0.450

S3 - Summary and details of the GLMM and pairwise post-hoc test, modeling how gait and limb type may affect spread.

[a] Summary of the full model - Influence of the fixed factors (Gait: Canter - Trot - Walk; Hoof: Fore - Hind) on the deviance. The horse is the random factor (horse ID).

[b] Results for the pairwise post-hoc test on the interaction gait * hoof

a

Generalized Linear Model
Family: beta
Spread ~ gait * hoof + (horse ID)
Full vs Null (Chisq = 16.984; df = 5; p = 0. 005)

Variable	Estimate	SE	z value	p-value (z)
(Intercept) [b,c]	-5.540	0.205	[a]	[a]
Gait (t) [b,c]	0.384	0.284	1.355	0.175
Gait (w) [b,c]	0.626	0.233	2.681	0.007
Hoof (h) [b,c]	0.637	0.254	2.509	0.012
Gait (t): hoof (h) [b,c]	-0.322	0.346	-0.930	0.352
Gait (w): hoof (h) [b,c]	-0.443	0.304	-1.454	0.146

[a] Not shown as not having a meaningful interpretation

[b] Estimate ± SE refer to the difference in the response between the reported level of this categorical predictor and the reference category of the same predictor

[c] Reference categories: "gait (c)", "hoof (f)"

b

***Post-hoc* comparisons**

Emmeans

Gait	Hoof	emmean	SE	lower.CL	upper.CL
canter	fore	-5.540	0.205	-5.940	-5.140
trot	fore	-5.160	0.221	-5.590	-4.720
walk	fore	-4.910	0.125	-5.160	-4.670
canter	hind	-4.900	0.154	-5.200	-4.600
trot	hind	-4.840	0.129	-5.090	-4.590
walk	hind	-4.720	0.116	-4.950	-4.490

Contrasts

Contrast	Estimate	SE	z ratio	p-value
c f - t f	-0.384	0.284	-1.355	0.754
c f - w f	-0.626	0.233	-2.681	0.079
c f - c h	-0.637	0.254	-2.509	0.122
c f - t h	-0.700	0.231	-3.026	0.030
c f - w h	-0.820	0.227	-3.611	0.004
t f - w f	-0.241	0.248	-0.971	0.927
t f - c h	-0.253	0.268	-0.944	0.935
t f - t h	-0.315	0.247	-1.275	0.799
t f - w h	-0.436	0.243	-1.794	0.470
w f - c h	-0.012	0.191	-0.060	1.000
w f - t h	-0.074	0.173	-0.427	0.998
w f - w h	-0.195	0.163	-1.191	0.841
c h - t h	-0.062	0.197	-0.317	1.000
c h - w h	-0.183	0.187	-0.977	0.925
t h - w h	-0.121	0.166	-0.725	0.979

S4 - Summary and details of the GLMM and pairwise post-hoc test modeling how gait may affect relative phase.

[a] Summary of the full model - Influence of the fixed factors (Gait: Canter - Trot - Walk) on the deviance. The horse is the random factor (horse ID).

[b] Results for the pairwise post-hoc test on the interaction gait

a				
**Generalized Linear Model**<br>Family: beta<br>Phase lag ~ gait + (horse ID)<br>Full vs Null (Chisq = 1258.103; df = 2; p < .0001)				
**Variable**	**Estimate**	**SE**	**z value**	**p-value (z)**
(Intercept) [b,c]	1.071	0.037	[a]	[a]
Gait (t) [b,c]	-1.006	0.017	-58.930	<2e-16
Gait (w) [b,c]	0.049	0.017	2.860	0.004
[a] Not shown as not having a meaningful interpretation<br>[b] Estimate ± SE refer to the difference in the response between the reported level of this categorical predictor and the reference category of the same predictor<br>[c] Reference categories: “gait (c)”				
b<br>***Post-hoc* comparisons**				
**Emmeans**				
**Gait**	**emmean**	**SE**	**lower.CL**	**upper.CL**
canter	1.071	0.037	0.998	1.144
trot	0.065	0.037	-0.007	0.136
walk	1.120	0.037	1.048	1.191
**Contrasts**				
**Contrast**	**Estimate**	**SE**	**z ratio**	**p-value**
c - t	1.006	0.017	58.934	<.0001
c - w	-0.049	0.017	-2.860	0.012
t - w	-1.055	0.015	-69.084	<.0001

S5 - Summary and details of the GLMM and pairwise post-hoc test modeling how Hind Fore Interval (HFI), gait and saddle may affect the Fore Hoof Interval (FHI).

[a] Summary of the full model - Influence of the fixed factors (Gait: Canter - Trot - Walk; Saddle: English - Side) on the deviance. The horse is the random factor (horse ID).

[b] Results for the pairwise post-hoc test on the interaction HFI * gait * saddle

a

Generalized Linear Model

Family: beta

FHI ~ HFI * gait * saddle + (horse ID)

Full vs Null (Chisq = 1036.630; df = 10; p < .0001)

Variable	Estimate	SE	z value	p-value (z)
(Intercept) [b,c]	-5.262	0.450	[a]	[a]
Inter [b,c]	7.696	1.025	7.505	<.001
Gait (t) [b,c]	2.619	0.466	5.617	<.00
Gait (w) [b,c]	1.336	0.468	2.855	0.004
Saddle (side) [b,c]	-0.605	0.572	-1.058	0.290
Inter: gait (t) [b,c]	-3.060	1.071	-2.856	0.004
Inter: gait (w) [b,c]	-2.192	1.034	-2.119	0.034
Inter: saddle (side) [b,c]	1.148	1.292	0.888	0.375
Gait (t): saddle (side) [b,c]	0.667	0.620	1.076	0.282
Gait (w): saddle (side) [b,c]	1.297	0.611	2.123	0.034
Inter: gait (t): saddle (side) [b,c]	-1.423	1.422	-1.001	0.317
Inter: gait (w): saddle (side) [b,c]	-1.958	1.319	-1.485	0.138

[a] Not shown as not having a meaningful interpretation

[b] Estimate ± SE refer to the difference in the response between the reported level of this categorical predictor and the reference category of the same predictor

[c] Reference categories: “gait (g)”, “saddle (english)”

b

***Post-hoc* comparisons**

Emmeans

Gait	Saddle	emmean	SE	lower.CL	upper.CL
canter	english	-0.646	0.172	-0.983	-0.310
trot	english	0.138	0.087	-0.032	0.308
walk	english	-0.625	0.053	-0.729	-0.522
canter	side	-0.563	0.136	-0.829	-0.297
trot	side	0.035	0.103	-0.167	0.236
walk	side	-0.420	0.061	-0.540	-0.300

Contrasts

Contrast	Estimate	SE	z ratio	p-value
c english - t english	-0.784	0.181	-4.332	<.0001
c english - w english	-0.021	0.177	-0.118	1.000
c english - c side	-0.083	0.209	-0.399	0.999
c english - t side	-0.681	0.189	-3.604	0.004
c english - w side	-0.226	0.180	-1.255	0.809
t english - w english	0.763	0.099	7.703	<.0001
t english - c side	0.701	0.148	4.729	<.0001
t english - t side	0.103	0.119	0.871	0.954
t english - w side	0.558	0.105	5.330	<.0001
w english - c side	-0.063	0.142	-0.442	0.998
w english - t side	-0.660	0.111	-5.957	<.0001
w english - w side	-0.205	0.063	-3.240	0.015
c side - t side	-0.598	0.160	-3.747	0.003
c side - w side	-0.143	0.145	-0.988	0.922
t side - w side	0.455	0.116	3.905	0.001
